# circ_HMGCS1 modulates hepatocellular carcinoma chemoresistance via miR‐338‐5p/IL‐7 pathway

**DOI:** 10.1111/jcmm.18137

**Published:** 2024-03-06

**Authors:** Siyu Zhang, Jun Ma, Tingdong Yu, Zhengrui Song, Wan Yee Lau, Yong Zha

**Affiliations:** ^1^ Department of Hepatobiliary and Pancreatic Surgery The Third Affiliated Hospital of Kunming Medical University, Yunnan Cancer Hospital Kunming China; ^2^ Department of Medical Oncology Sichuan Clinical Research Center for Cancer, Sichuan Cancer Hospital & Institute, Sichuan Cancer Center Affiliated Cancer Hospital of University of Electronic Science and Technology of China Chengdu China; ^3^ Faculty of Medicine The Chinese University of Hong Kong Hong Kong SAR China

**Keywords:** circ_HMGCS1/miR‐338‐5p/IL‐7, cisplatin chemoresistance, hepatocellular cancer, immunotherapy

## Abstract

Hepatocellular cancer is one of the most serious types of cancer in the world, with high incidence and mortality rates. Most HCC patients with long‐term chemotherapy develop chemoresistance, leading to a poor prognosis. However, the underlying mechanism of circRNAs in HCC chemoresistance remains unclear. Our research found that circ_0072391(circ_HMGCS1) expression was significantly upregulated in cisplatin‐resistant HCC cells. The silence of circ_HMGCS1 attenuated the cisplatin resistance in HCC. Results showed that circ_HMGCS1 regulated the expression of miR‐338‐5p via acting as microRNA sponges. Further study confirmed that miR‐338‐5p regulated the expression of IL‐7. IL‐7 could remodel the immune system by improving T‐cell function and antagonising the immunosuppressive network. IL‐7 is an ideal target used to enhance the function of the immune system. circ_HMGCS1 exerts its oncogenic function through the miR‐338‐5p/IL‐7 pathway. Inhibition of circ_HMGCS1/miR‐338‐5p/IL‐7 could effectively attenuate the chemoresistance of HCC. IL‐7 might be a promising immunotherapy target for HCC cancer treatment.

## 1 INTRODUCTION

Hepatocellular cancer is one of the most serious types of cancer in the world, with high incidence and mortality rates.^1^, ^2^ Hepatocellular carcinoma (HCC) is the fifth most common cancer and the second leading cause of cancer death worldwide.^3^ Surgical resection with chemotherapy is curative for the early stage of HCC.^4^, ^5^ Unfortunately, most HCC patients are usually diagnosed at an advanced stage because of the lack of diagnosis at an early stage.^6^ In the past decades, surgical techniques, targeted therapy and immunotherapy have developed rapidly. However, the 5‐year overall survival rate of HCC patients is approximately 14% due to metastasis and chemoresistance.^7^, ^8^, ^9^ Most HCC patients with long‐term chemotherapy develop chemoresistance, leading to a poor prognosis.^9^, ^10^, ^11^ Cisplatin (CDDP) is the main drug for HCC patients, but the specific mechanism underlying the biological mechanisms of CDDP resistance in HCC remains unclear.^12^, ^13^ Therefore, further exploration of chemoresistance biomarkers and mechanisms will provide new, promising therapeutic strategies for HCC.

Circular RNAs (circRNAs) are a circular type of non‐coding RNAs (ncRNAs) with important biological functions. circRNAs are characterised by a closed‐loop structure with a 5′ cap and 3′ polyadenylation tail covalently connected.^14^, ^15^ CircRNAs are more stable and resistant to RnaseR than linear RNAs due to this special loop structure.^16^ circRNA is derived from the precursor mRNA back splice in transcription.^17^ More research has confirmed that circRNAs were able to regulate gene expression at both transcriptional by acting as “sponges” for microRNAs (miRNAs).^18^, ^19^ With the development of bioinformatics and sequencing technologies, increasingly circRNAs are found and reported to play important roles in various biological activities.^20^ An increasing amount of evidence shows that circRNAs play an important role in HCC occurrence and development.

To figure out the underlying mechanism of HCC cisplatin, we generated a cisplatin HCC cell line by long‐time cisplatin treatment. We analysed the RNA sequencing database from GEO datasets (GEO: GSE159220, GSE114564 and GSE77509),^21^ and selected the upregulated circRNAs in HCC tissues. The GEO datasets all showed that circ_0002130, circ_0072391, circ_0000524, circ_0000384, circ_0003056, circ_0002566, circ_0072309 and circ_0004458 were significantly different expressed in cancer cells and normal cells. We performed experiments to detect the expression and function of these circRNAs in HCC cisplatin resistance. The results showed that circ_0072391(circ_HMGCS1) was significantly upregulated in cisplatin‐resistant HCC cell lines. The circular RNA interactome database shows that circ_0072391 is derived from the HMGCS1 Gene. circ_HMGCS1 has 331 bps in length and is located in chr5:43295853–43297268.

We hypothesised that circ_HMGCS1 might play an important role in HCC cisplatin resistance. To investigate the function of circ_HMGCS1, we overexpressed circ_HMGCS1 in HCC cell lines and silenced circ_HMGCS1 in HCC cisplatin‐resistant cell lines. Biological experiments were applied to investigate the downstream signal pathway. The results showed that circ_HMGCS1 upregulated the expression of interleukin‐7 (IL‐7) by acting as microRNA sponges for miR‐338‐5p. IL‐7 is one of the multipotent cytokines that maintain immune system homeostasis. IL‐7 plays a critical role in B cell and T cell proliferation, development and difference.^21^ IL‐7 is reported to be closely associated with the progression of cancer and has been used as a target in cancer immune therapy. Our results showed that circ_HMGCS1 might be a novel mediator in HCC cisplatin resistance, as well as a novel immune therapeutic target.

## 2 MATERIALS AND METHODS

### 2.1 Cell culture

Human HCC cell lines, HepG2 and SMMC‐7721, were cultured in DMEM (Gibco, Carlsbad, CA, USA) supplemented with 10% FBS (Gibco, Carlsbad, CA, USA). HCC cell lines were obtained from the Cell Bank of Type Culture Collection of the Chinese Academy of Sciences (Shanghai Institute of Cell Biology). All cell lines were cultured in humidified incubators with 5% CO_2_ at 37°C.

### 2.2 RNA extraction and reverse transcription

TRIzol (Invitrogen, Carlsbad, CA, USA) was applied to extract total RNA from cell lines following the manufacturer's instructions. cDNAs were synthesised from 1 μg total RNA by using the Superscript III transcriptase (Invitrogen, Carlsbad, CA, USA).

### 2.3 Quantitative real‐time PCR analysis

qRT‐PCR was performed on an ABI 7300 qPCR system with SYBR green. SYBR Green PCR Master Mix (Applied Biosystem, Foster City, CA, USA) was used. Relative expression was normalised to GAPDH. The primers were shown in Table S1. Relative gene expressions were calculated using the $2^{-∆∆C_{t}}$ method.

### 2.4 RNase R treatment

RNase R treatment was performed with 4 U/mg RNase R (Epicentre Technologies, Madison, WI, USA) for 30 min at 37°C. qRT‐PCR was applied to detect the changes in RNA levels.

### 2.5 Actinomycin D (ActD) assay

Hepatocellular carcinoma cells were cultured with 2 μg/mL ActD (Sigma‐Aldrich, St. Louis, MO, USA) and collected at 0, 2, 6, 12 and 24 h. The relative RNA levels were detected by qRT‐qPCR.

### 2.6 Nucleocytoplasmic separation

The RNA of cytoplasmic and nuclear fractions was separated by using a Paris kit (Invitrogen, Carlsbad, CA, USA) following the manufacturer's instructions. The relative RNA levels were detected by qRT‐qPCR.

### 2.7 Western blot

Hepatocellular carcinoma cells were collected and lysed with RIPA buffer with proteinase inhibitor. BCA Protein Assay Kit (Beyotime, China, Shanghai) was applied to detect the concentration of protein. SDS‐PAGE was used to separate the proteins. 25 μg proteins were separated by gel electrophoresis. The proteins were transferred to PVDF membranes (Millipore, Billerica, MA, USA). After blocking with 5% nonfat milk, specific primary antibodies were used to incubate membranes. Then, the membranes were incubated with HRP‐conjugated secondary antibodies and detected by using the ECL system (Thermo Fisher Scientific, Rochester, NY, USA).

### 2.8 Luciferase reporter assay

The wild‐type or mutant sequences of circRNAs were inserted into PGL3 plasmids. HCC cells were seeded in 24‐well plates with 3 × 10^4^ cells. Lipofectamine 3000 (Invitrogen) was used for transfections of luciferase plasmids. HCC cells were transfected with wild‐type or mutant circ_HMGCS1 and miR‐338‐5P or NC mimics. 48 h after transfections, Dual‐Luciferase Assay (Promega, Madison, WI, USA) was applied for the detection of luciferase activity. The Firefly‐Luciferase activity was normalised to Renilla‐Luciferase activity.

### 2.9 IC50 analysis

Hepatocellular carcinoma cells were seeded into 96‐well plates with 6000 cells per well and cultured overnight. Then, the cells were treated with 0, 1, 2.5, 5, 10, 15, 25 and 50 mg/mL cisplatin, respectively. CCK8 kits were used to detect cell viability with an absorbance of 450 nm. IC50 was calculated based on the cell viability by using Prism 5.

### 2.10 RNA pull‐down

The RNA pull‐down assay was performed as reported previously. The biotinylated circ_HMGCS1 probe and the biotinylated negative control (NC) probe (GenePharma, China) were incubated with streptavidin magnetic beads (Invitrogen, USA) for 2 h at room temperature.1 × 10^7^ HCC cells were collected and lysed, then incubated with beads overnight at 4°C. Subsequently, eluted the beads bound with RNA complexes. The RNeasy Mini Kit (QIAGEN) was used to extract RNAs. The levels of RNAs were detected by qRT‐PCR.

### 2.11 Statistical analyses

All data are shown as the mean ± SD. The differences between the two groups were analysed by two‐tailed unpaired or paired Student's *t*‐test. The differences among multiple groups were analysed by one‐way or two‐way analysis of variance (ANOVA). *p* < 0.05 was considered statistically significant. ***p < 0.001, **p < 0.01, *p < 0.05.

## 3 RESULTS

### 3.1 Long‐time cisplatin treatments induce resistance in HCC

**FIGURE 1:**
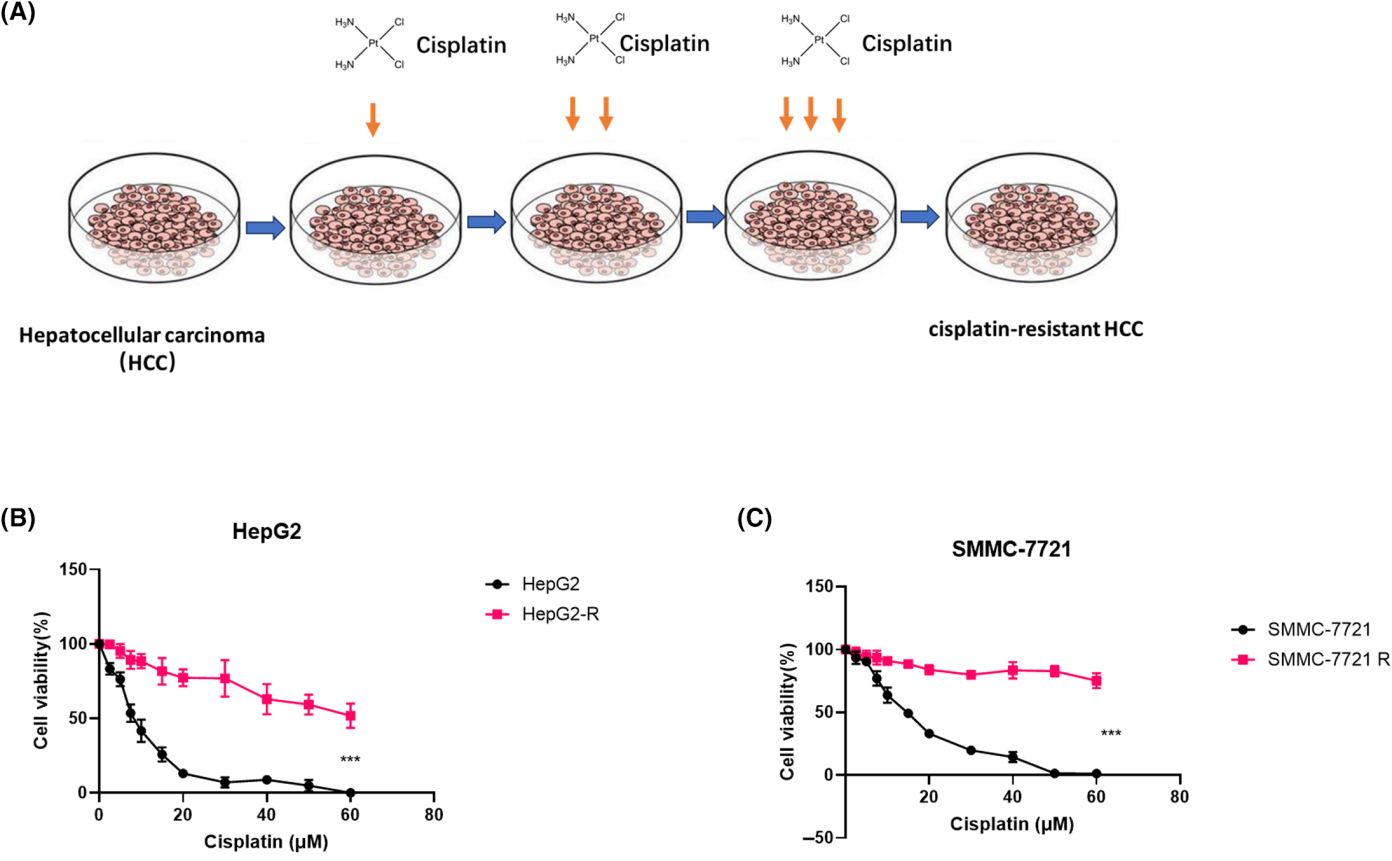
Identification of cisplatin‐resistant HCC cell lines. (A) The generations cisplatin‐resistant HCC cell lines. (B) CCK8 assays were performed to detect IC50 of cisplatin in HepG2 and HepG2‐R. (C) CCK8 assays were performed to detect IC50 of cisplatin in SMMC‐7721 and SMMC‐7721 R. ***p < 0.001.

Cisplatin therapy is recommended for patients with advanced HCC. Unfortunately, cisplatin resistance would inevitably develop with long‐term chemotherapy.^21^ Acquired chemoresistance is one of the main factors that lead to poor survival. Chemoresistance development is a very complex process. To investigate the underlying mechanism of chemoresistance in HCC, we generated a cisplatin‐resistant HCC cell line by long‐time treatment of cisplatin (Figure 1A). After cisplatin treatment of 3 months, CCK8 assays were used to detect the IC50 of cisplatin in HepG2 (Figure 1B) and SMMC‐7721 (Figure 1C). The results showed that HepG2 R had a higher IC50 than HepG2 (Figure 1B). SMMC‐7721R had a higher IC50 than SMMC‐7721 (Figure 1C). The results showed that long‐time cisplatin treatments induce resistance in HCC.

### 3.2 circ_HMGCS1 is upregulated in cisplatin‐resistant HCC cell lines

**FIGURE 2:**
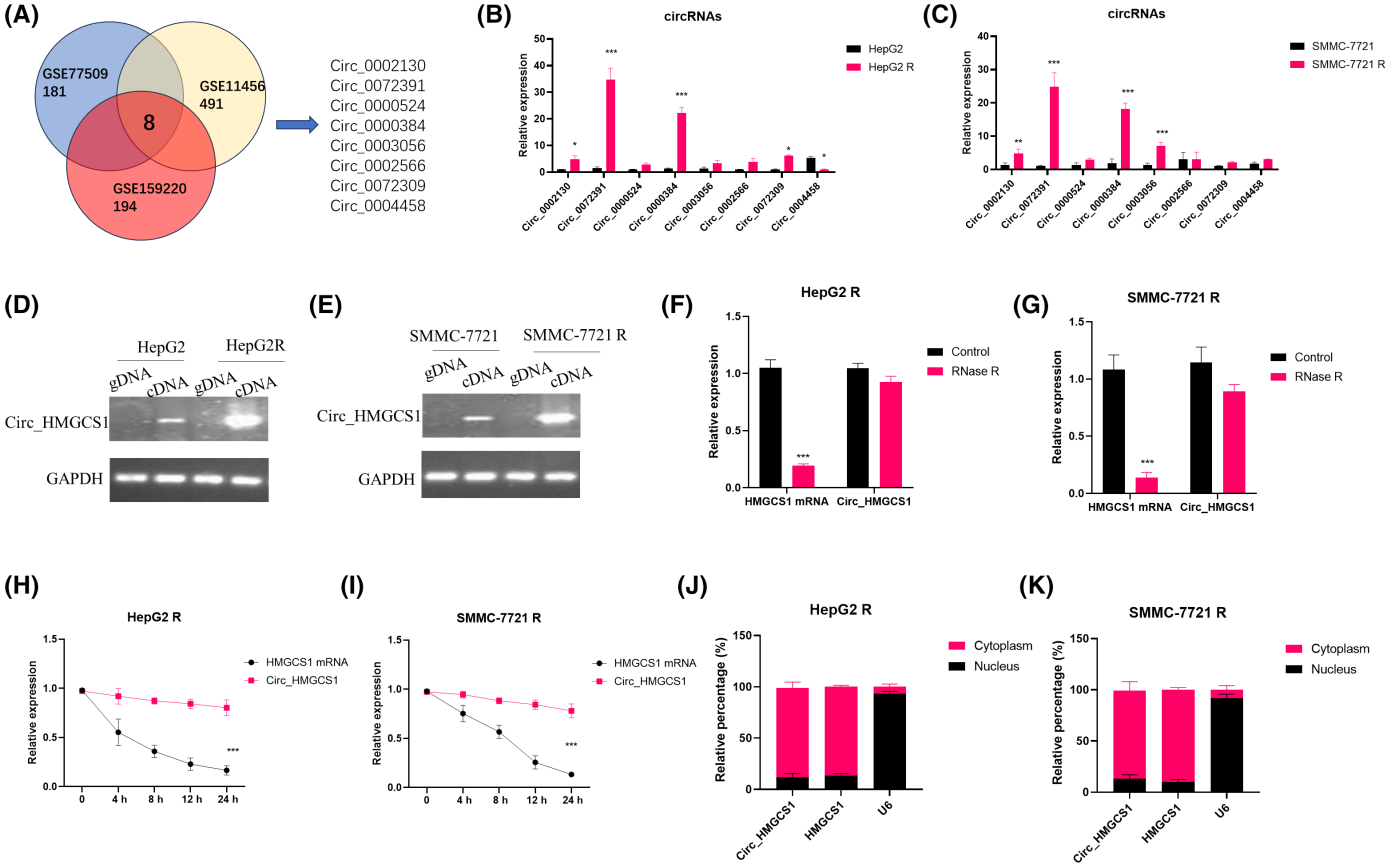
circ_HMGCS1 is upregulated in cisplatin‐resistant HCC cell lines. (A) Schematic illustration shows the significantly different expressions of circRNAs predicted by overlapping GEO: GSE114564, GSE77509 and GSE159220 data. (B) qRT‐PCR was used to detect the expressions of predicted circRNAs in HepG2 and HepG2 R. (C) qRT‐PCR was used to detect the expressions of predicted circRNAs in SMMC‐7721 and SMMC‐7721 R. (D) RT‐PCR was performed in gDNA and cDNA from HepG2 and HepG2R. (E) RT‐PCR was performed in gDNA and cDNA from SMMC‐7721 and SMMC‐7721 R. (F, G) RNase R assays were performed in HepG2 R (F) and SMMC‐7721 R (G). (H, I) Actinomycin D Assays were performed to detect the half‐life of circ_HMGCS1 and HMGCS1 mRNA. (J, K) Nuclear and cytoplasm extraction experiments were performed in HepG2 R (J) and SMMC‐7721 R (K), and qRT‐PCR was used to detect the expressions of circ_HMGCS1 and HMGCS1 mRNA. ***p < 0.001, **p < 0.01, *p < 0.05.

To investigate the function of circRNAs in HCC progression, we mined three GEO RNA sequence databases (GEO: GSE159220, GSE114564 and GSE77509). We selected eight significantly differentially expressed circRNAs in the intersection of three databases for further research (Figure 2A). We detected the expressions of 8 circRNAs in both parent and cisplatin‐resistant HCC cell lines (Figure 2B,C). The results showed that circ_0072391(circ_HMGCS1) and circ_0000384 were significantly upregulated in HepG2R (Figure 2B) and SMMC‐7721R (Figure 2C). circ_HMGCS1 had the largest upregulation ratio during the progression of acquiring chemoresistance. The expressions of circ_HMGCS1 were also identified by RT‐PCR in gDNA/cDNA from HepG2/HepG2 R (Figure 2D) and SMMC‐7721/SMMC‐7721R (Figure 2E). RNase R Assay and Actinomycin D Assay were performed in HepG2 R and SMMC‐7721 R cells to detect the stabilities of circ_HMGCS1 and HMGCS1 mRNA (Figures 2F–I). Results showed that circ_HMGCS1 was more resistant to RNase R digestion in HepG2R (Figure 2F) and SMMC‐7721R (Figure 2G). circ_HMGCS1 had longer half‐life than HMGCS1 mRNA (Figure 2H,I). Nuclear and cytoplasm extraction experiments showed that most of circ_HMGCS1 is located in cytoplasm, indicating that circ_HMGCS1 might regulate the target after transcription (Figure 2J,K).

### 3.3 circ_HMGCS1 contributes to HCC cisplatin resistance

**FIGURE 3:**
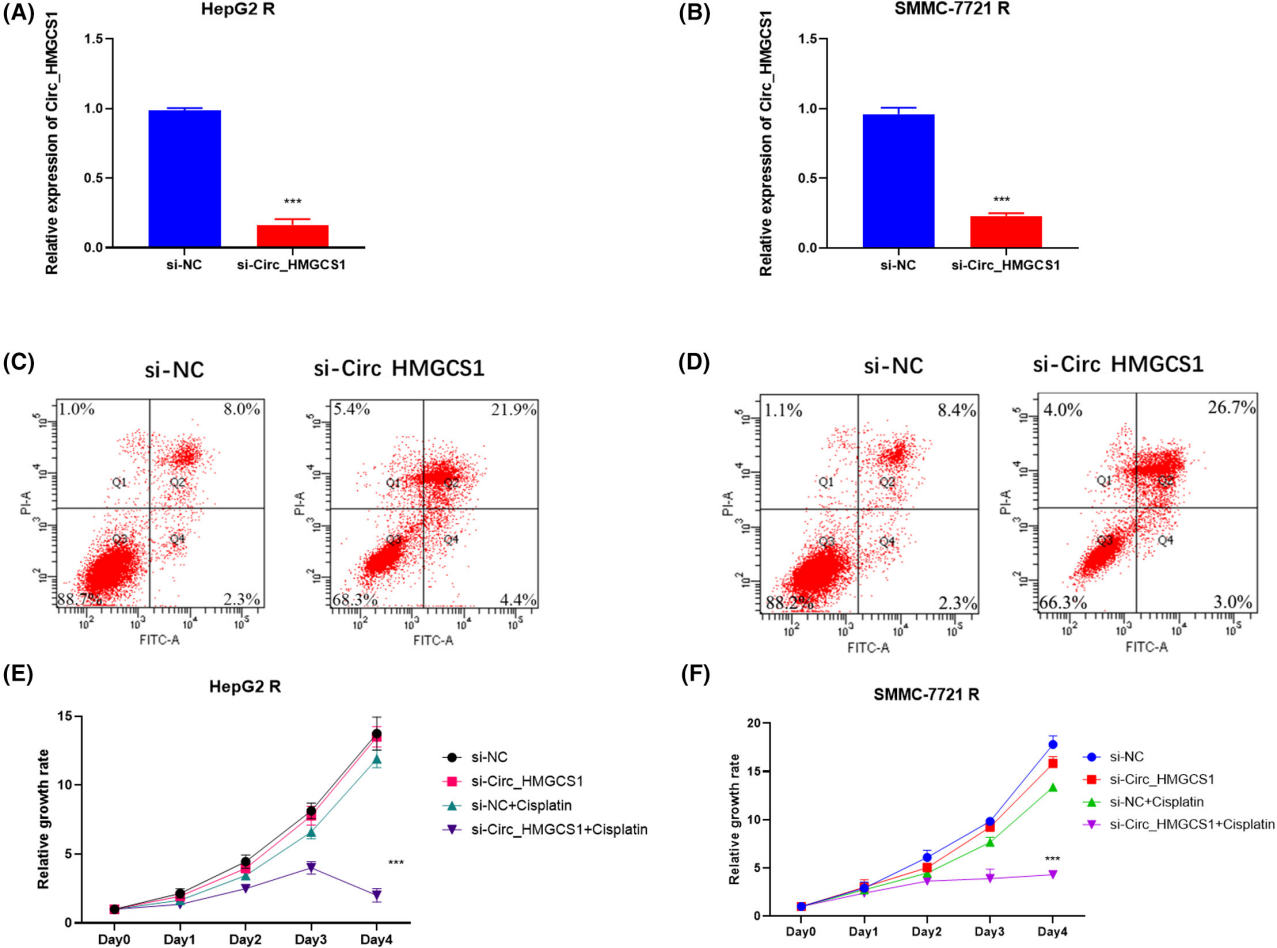
circ_HMGCS1 contributes to HCC cisplatin resistance. (A) qRT‐PCR was used to detect the expression of circ_HMGCS1 in HepG2 R cells. (B) qRT‐PCR was used to detect the expression of circ_HMGCS1 in SMMC‐7721 R cells. (C) Apoptosis assay was used to detect apoptosis in HepG2 R‐NC and si‐circ_HMGCS1. (D) Apoptosis assay was used to detect apoptosis in SMMC‐7721 R‐NC and si‐circ_HMGCS1. (E) CCK8 assay was performed in HepG2R NC and si‐circ_HMGCS1 cells with the treatment of cisplatin. (F) CCK8 assay was performed in SMMC‐7721R NC and si‐circ_HMGCS1 cells with the treatment of cisplatin. ***p < 0.001.

To investigate the function of circ_HMGCS1 in HCC cisplatin resistance, we silenced circ_HMGCS1 in HepG2 R (Figure 3A) and SMMC‐7721 R (Figure 3B). Apoptosis assays and CCK8 assays were used to detect the influence of circ_HMGCS1 on HCC cisplatin resistance. Apoptosis results showed that the silence of circ_HMGCS1 significantly increased the apoptosis rate of HepG2R (Figure 3C) and SMMC‐7721R (Figure 3D). CCK8 assay was used to detect the influence of circ_HMGCS1 on cisplatin‐induced growth inhibition (Figure 3E,F). The results showed that cisplatin treatment did not influence the growth of HepG2 R cells, while cisplatin inhibited HepG2 R growth with silence of circ_HMGCS1 (Figure 3E). Similar results were obtained from SMMC‐7721 R cells (Figure 3F). These results indicated that circ_HMGCS1 contributes to HCC cisplatin resistance.

### 3.4 circ_HMGCS1 serves as a sponge for miR‐338‐5p

**FIGURE 4:**
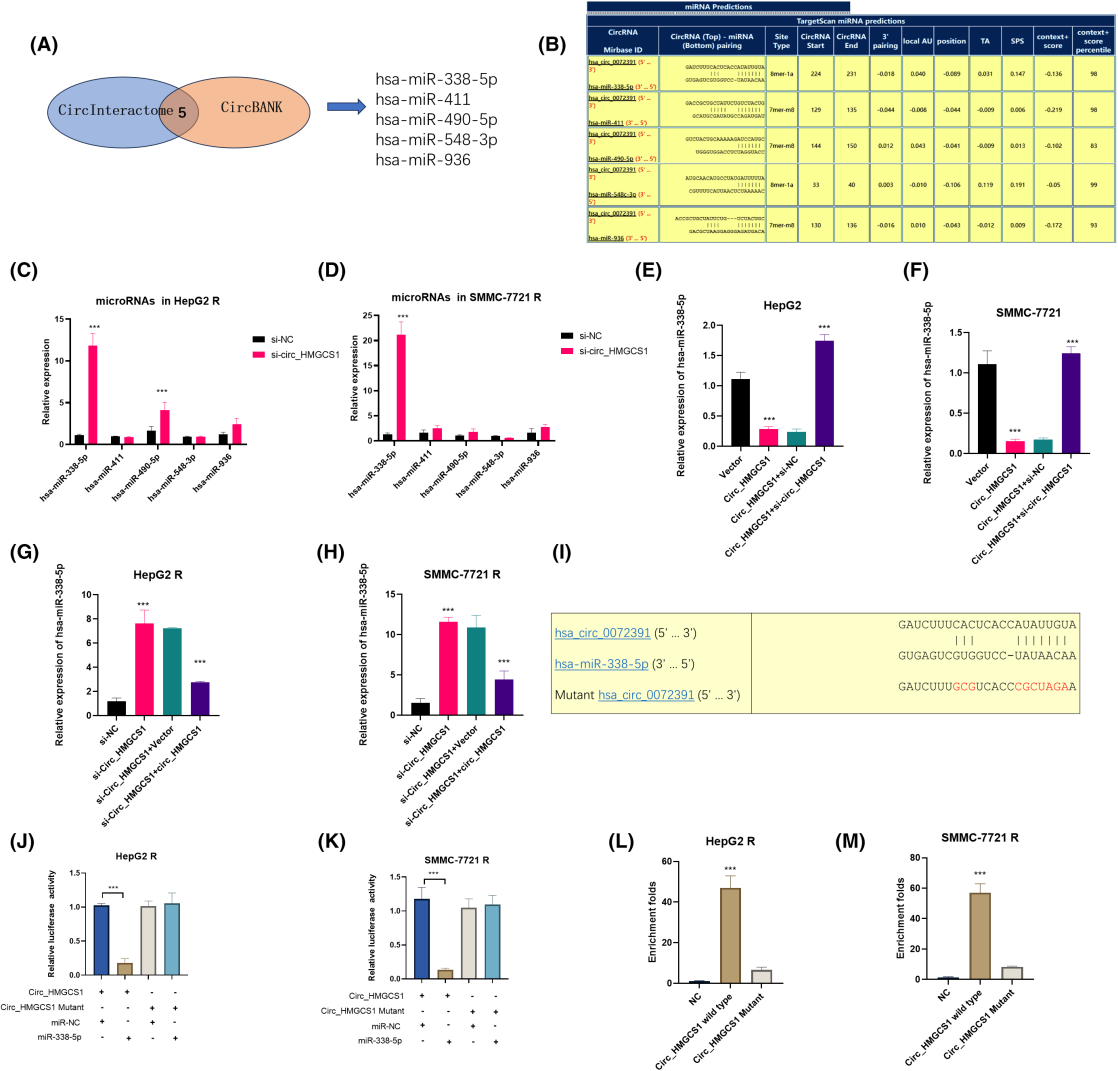
circ_HMGCS1 serves as a sponge for miR‐338‐5p. (A) Schematic illustration of the target miRNAs of circ_HMGCS1 predicted by overlapping CircInteractome and circBank databases. (B) Detailed information of predicted miRNAs. (C) qRT‐PCR assay was used to detect the expression of predicted miRNAs in HepG2 R NC and si‐circ_HMGCS1 cells. (D) qRT‐PCR assay was used to detect the expression of predicted miRNAs in SMMC‐7721 R NC and si‐circ_HMGCS1 cells. (E) qRT‐PCR assay was performed to detect the levels of miR‐338‐5p in HepG2 cells. (F) qRT‐PCR assay was performed to detect the levels of miR‐338‐5p in SMMC‐7721 cells. (G) qRT‐PCR assay was performed to detect the levels of miR‐338‐5p in HepG2 R cells. (H) qRT‐PCR assay was performed to detect the levels of miR‐338‐5p in SMMC‐7721 R cells. (I) The mutated sequence of the binding site in circ_HMGCS1. (J) Luciferase activity was performed in HepG2 R. (K) Luciferase activity was performed in SMMC‐7721 R. (L) RNA pull‐down assay was performed in HepG2 R. (M) RNA pull‐down assay was performed in SMMC‐7721 R. ***p < 0.001, **p < 0.01, *p < 0.05.

circRNAs could act as microRNA (miRNA) sponges by binding to complementary miRNA and regulating the expression of miRNA.^17^, ^22^ We further performed experiments to investigate whether circ_HMGCS1 enhances HCC cisplatin resistance through interacting with miRNAs. CircInteractome and circBank bioinformatics databases were applied to predict the potential miRNAs. Five microRNAs were predicted potential targets in both databases (Figure 4A). The predicted binding sites are shown in Figure 4B. We first detected the expression of 5 miRNAs in HepG2 R NC/si‐circ_HMGCS1 (Figure 4C) and SMMC‐7721 NC/si‐circ_HMGCS1 (Figure 4D). The results showed that the silence of circ_HMGCS1 could significantly upregulate the expression of miR‐338‐5p (Figure 4C,D). We then confirmed the expression of miR‐338‐5p with rescue experiments. The results indicated that miR‐338‐5p was downregulated in circ_HMGCS1 overexpression cell lines and upregulated with silence of circ_HMGCS1(Figure 4E,F). And miR‐338‐5p was significantly upregulated in circ_HMGCS1 silenced cisplatin‐resistant HCC cell lines and downregulated with reexpression of circ_HMGCS1 (Figure 4G,H). We mutated the binding site in circ_HMGCS1 and constructed pGL3 luciferase plasmids containing sequences of wild‐type circ_HMGCS1 or mutant circ_HMGCS1 (Figure 4I). Luciferase activity assay results showed that miR‐338‐5p could downregulate the luciferase activity of pGL3‐WT‐circ_HMGCS1, while miR‐338‐5p had little influence on the luciferase activity of pGL3‐Mutant‐circ_HMGCS1 in HepG2R (Figure 4J) and SMMC‐7721R (Figure 4K). To confirm whether circ_HMGCS1 and miR‐338‐5p have direct interaction, we carried out RNA pull‐down assays in HepG2 R and SMMC‐7721R. The results showed that wild‐type circ_HMGCS1 had a direct interaction with miR‐338‐5p, while mutation of the binding site abrogated the interaction in HepG2 R (Figure 4L). Similar results were obtained in SMMC‐7721R (Figure 4M). All these results confirmed that circ_HMGCS1 had a direct interaction with miR‐338‐5p and regulated the expression of miR‐338‐5p.

### 3.5 miR‐338‐5p regulates IL‐7 expression

**FIGURE 5:**
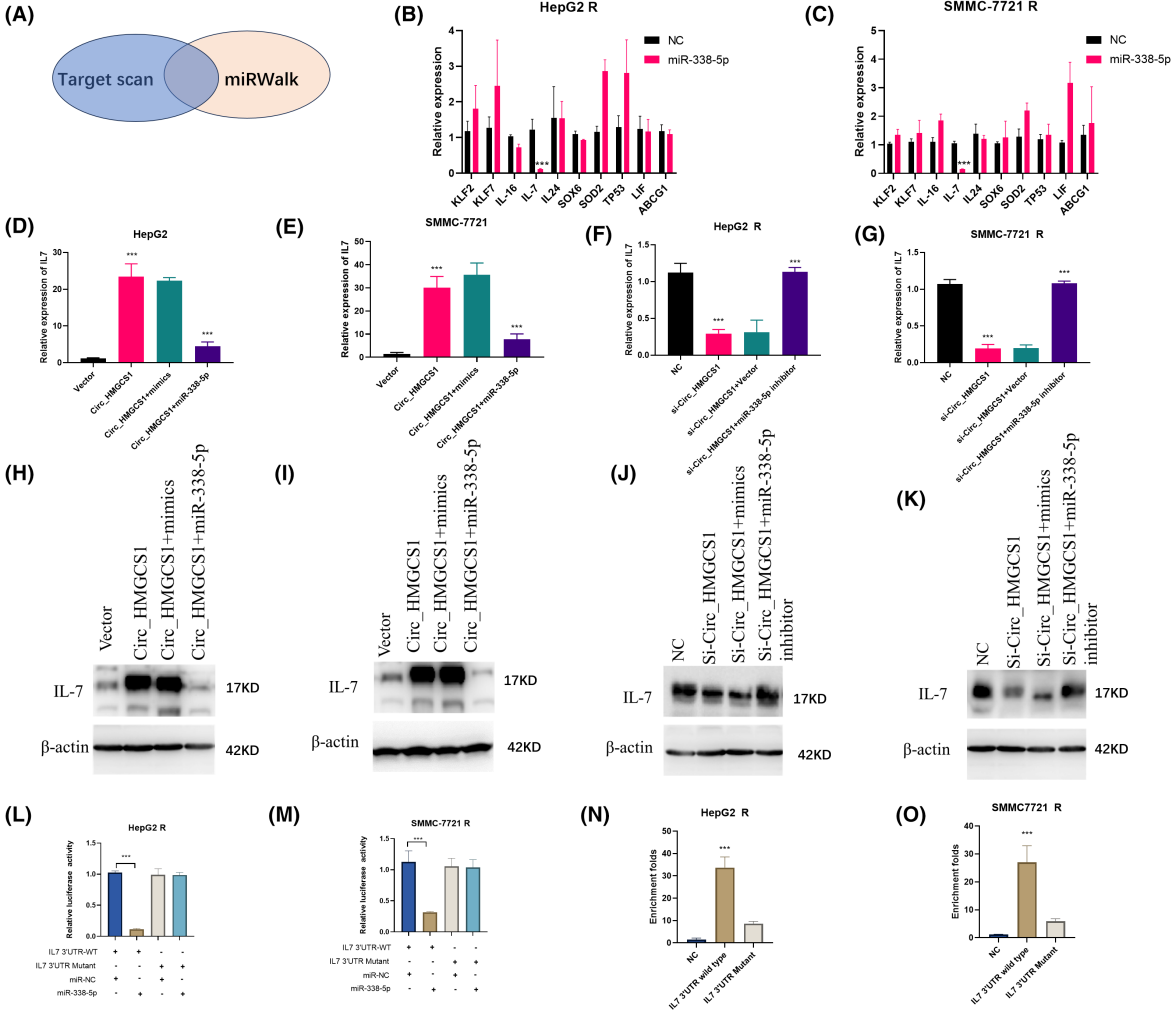
circ_HMGCS1 sponges miR‐338‐5p to regulate the expression of IL‐7 in HCC cells. (A) Schematic illustration showing the predicted target genes by overlapping miRWalk and Targetscan. (B) qRT‐PCR was used to detect the predicted mRNAs in HepG2 R NC and miR‐338‐5p cells. (C) qRT‐PCR was used to detect the predicted mRNAs in SMMC‐7721 R NC and miR‐338‐5p cells. (D) qRT‐PCR was used to detect the mRNA of IL‐7 in HepG2 cells. (E) qRT‐PCR was used to detect the mRNA of IL‐7 in SMMC‐7721 cells. (F) qRT‐PCR was used to detect the mRNA of IL‐7 in HepG2R cells. (G) qRT‐PCR was used to detect the mRNA of IL‐7 in SMMC‐7721R cells. (H) Western blot was used to detect the protein level of IL‐7 in HepG2 cells. (I) Western blot was used to detect the protein level of IL‐7 in SMMC‐7721 cells. (J) Western blot was used to detect the protein level of IL‐7 in HepG2 R cells. (K) qRT‐PCR was used to detect the protein level of IL‐7 in SMMC‐7721R cells. (L) Luciferase activity assays were performed in HepG2 R cells. (M) Luciferase activity assays were performed in SMMC‐7721 R cells. (N) RNA pull‐down assays were performed in HepG2 R cells. (O) RNA pull‐down assays were performed in SMMC‐7721R R cells. ***p < 0.001, **p < 0.01, *p < 0.05.

By using Target scan and miRWalk, we screened out ten downstream targets of miR‐338‐5p (Figure 5A). We performed qRT‐PCR to detect the expressions of predicted proteins in HepG2 R and SMMC‐7721R with transfections of miR‐338‐5p (Figure 5B,C). The results showed that transfection of miR‐338‐5p could effectively inhibit the expression of IL‐7 (Figure 5B,C). To confirm the regulation of miR‐338‐5p on IL‐7, we carried out rescue experiments in HepG2 /HepG2 R and SMMC‐7721/SMMC‐7721 R (Figures 5D–G). Transfection of circ_HMGCS1 increased the expression of IL‐7mRNA, and transfection of miR‐338‐5p into circ_HMGCS1 overexpression cells effectively blocked the upregulation of IL‐7 mRNA (Figures 5D,E). Silence of circ_HMGCS1 could inhibit the expression of IL‐7 mRNA, and transfection of miR‐338‐5p inhibitor could rescue the expression of IL‐7 mRNA (Figure 5F,G). Western blot was used to detect the influence of circ_HMGCS1 and miR‐338‐5p on IL‐7 protein level. Results showed that circ_HMGCS1 and miR‐338‐5p could regulate the protein level of IL‐7 (Figures 5H–K). To further confirm the direct binding between miR‐338‐5p and IL‐7 mRNA, we performed luciferase activity assays and RNA pull‐down assays. The results indicated that miR‐338‐5p could decrease the luciferase activity of pGL3‐IL‐7‐3'UTR‐wild type, but miR‐338‐5p could not influence the luciferase activity of pGL3‐IL‐7‐3'UTR‐mutated in HepG2 R and SMMC‐7721 R (Figure 5L,M). RNA pull‐down assay showed that miR‐338‐5p had direct interaction with wild‐type IL‐7‐3'UTR, while mutation of binding sites abrogated the interaction between them (Figure 5N,O). These results showed that circ_HMGCS1 could regulate the protein level of IL‐7 through miR‐338‐5p.

### 3.6 circ_HMGCS1/ miR‐338‐5p/IL‐7 pathway contributed to HCC cisplatin resistance

**FIGURE 6:**
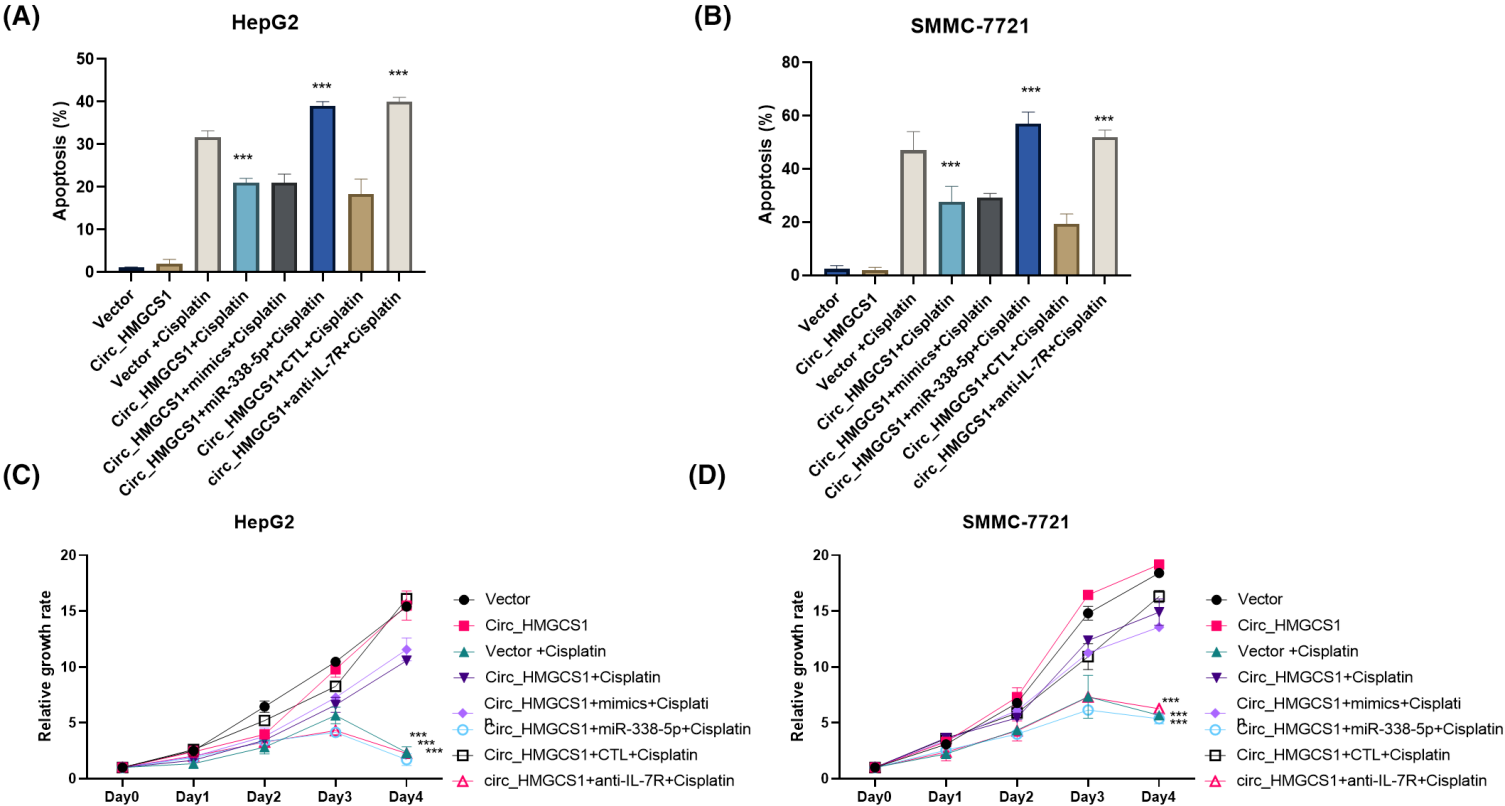
circ_HMGCS1/ miR‐338‐5p/IL‐7 pathway contributed to HCC cisplatin resistance. (A) Apoptosis assays were performed to detect apoptosis in HepG2 with indicated treatment. (B) Apoptosis assays were performed to detect apoptosis in SMMC‐7721 with indicated treatment. (C) CCK8 assays were performed to detect proliferation in HepG2 with indicated treatment. (D) CCK8 assays were performed to detect proliferation in SMMC‐7721 with indicated treatment. ***p < 0.001, **p < 0.01, *p < 0.05.

In our research, we confirmed the influence of circ_HMGCS1/miR‐338‐5p on IL‐7 expression. We inhibited miR‐338‐5p/IL‐7 pathway in HepG2 circ_HMGCS1 overexpression cells. The results showed that circ_HMGCS1 overexpression could decrease the cisplatin‐induced apoptosis, and transfection of miR‐338‐5p or treatment with IL‐7R antibody could effectively block the reduction of apoptosis induced by circ_HMGCS1 overexpression and increase apoptosis rate (Figure 6A). The experiments were carried out in SMMC‐7721, and similar results confirmed the conclusion (Figure 6B). We further performed a CCK8 assay to detect the influence of circ_HMGCS1/ miR‐338‐5p/IL‐7 on cisplatin‐induced proliferation inhibition. Results showed that circ_HMGCS1 overexpression could increase the proliferation of HepG2 with the treatment of cisplatin, and transfection of miR‐338‐5p or treatment with IL‐7R antibody could effectively block the proliferation induced by circ_HMGCS1 overexpression (Figure 6C). The experiments were carried out in SMMC‐7721, and similar results confirmed the conclusion (Figure 6D). All these results showed that the circ_HMGCS1/miR‐338‐5p/IL‐7 pathway contributed to HCC cisplatin resistance.

## 4 DISCUSSION

Advanced HCC patients are not candidates for local or regional treatment; they have a very limited amount of therapy.^23^ Chemotherapy is essential for HCC treatment, especially for advanced HCC patients.^23^, ^24^ Cisplatin is widely used in HCC chemotherapy, but chemoresistance significantly limits cisplatin curative effects. Cisplatin resistance acquisition becomes one of the vital factors in the poor prognosis of HCC.

In our research, the results showed that circ_HMGCS1 was significantly upregulated in HCC cisplatin‐resistant cells. circ_HMGCS1 was resistant to RNase R digestion and had a longer half‐life time than its linear isoform, which suggested that circ_HMGCS1 might be a valuable biomarker or target for HCC. Further study showed that circ_HMGCS1 could regulate the expression level of miR‐338‐5p by acting as microRNA sponges. Bioinformatic prediction and biological experiments confirmed that miR‐338‐5p could target IL‐7 and regulate its expression in HCC. circ_HMGCS1 regulates the expression of IL‐7 through miR‐338‐5p. Inhibition of circ_HMGCS1/miR‐338‐5p/IL‐7 pathway could attenuate the cisplatin resistance of HCC.

Interleukin‐7 (IL‐7) is a non‐haematopoietic cell‐derived cytokine with important functions in the adaptive immune system. What's more, IL‐7 contributes to lymph nodes (LN) organogenesis and activated T cells maintenance. Moreover, immunotherapy has emerged as a promising area in recent years, which utilises the immune system of patients themselves. IL‐7 is an ideal target used to enhance the function of the immune system.^25^ IL‐7 could remodel the immune system by improving T cell function and antagonising the immunosuppressive network.^26^

The IL‐7 has been reported to be essential in cancer progression. The expressions of IL‐7 is upregulated in various cancers such as prostate and breast cancer. Upregulation of IL‐7 is reported to be closely related to poor prognosis.^27^, ^28^ In our research, we found that circ_HMGCS1 could regulate the expression of IL‐7 through miR‐338‐5p. It is a new regulation for IL‐7 expression. And IL‐7 contributed to the cisplatin resistance of HCC. In future work, we will perform more experiments to investigate the application of IL‐7 as an immunotherapy for HCC cancer treatment and the underlying mechanism.

In summary, we confirmed the function of circ_HMGCS1 in HCC cisplatin resistance through bioinformatics and biological experiments. circ_HMGCS1 exerts its oncogenic function through miR‐338‐5p/IL‐7 pathway. Inhibition of circ_HMGCS1/miR‐338‐5p/IL‐7 could effectively attenuate the chemoresistance of HCC. IL‐7 might be a promising immunotherapy target for HCC cancer treatment.

## AUTHOR CONTRIBUTIONS

**Siyu Zhang:** Conceptualization (equal); data curation (lead); formal analysis (equal); project administration (lead). **Jun Ma:** Formal analysis (equal); investigation (equal); methodology (equal); project administration (equal). **Tingdong Yu:** Resources (equal); software (equal); supervision (equal). **Zhengrui Song:** Validation (equal); visualization (equal). **Wan Yee Lau:** Funding acquisition (equal); software (equal); supervision (equal). **Yong Zha:** Supervision (equal); validation (equal); visualization (equal); writing – original draft (equal).

## FUNDING INFORMATION

This study was supported by the Lau WY Academician Workstation of Yunnan Province Science and Technology Platform Program (202305AF150067).

## CONFLICT OF INTEREST STATEMENT

The authors declare that they have no competing interests.

## Supporting information


Table S1.

## Data Availability

The datasets used are available on reasonable request.
