# Supplementary material for: circ_HMGCS1 modulates hepatocellular carcinoma chemoresistance via miR‐338‐5p/IL‐7 pathway
Source: J Cell Mol Med. 2024 Mar 6;28(6):e18137. doi: 10.1111/jcmm.18137 (PMC10915820; doi:10.1111/jcmm.18137)
Supplement: Supplementary file 1 — Table S1. [file JCMM-28-e18137-s001.docx]

| **GeneName** | **Primers Right** | **Primers Left** |
| --- | --- | --- |
| KLF2 | TTCGGTCTCTTCGACGACG | TGCGAACTCTTGGTGTAGGTC |
| KLF7 | AGACATGCCTTGAATTGGAACG | GGGGTCTAAGCGACGGAAG |
| IL-16 | GCCGAAGACCCTTGGGTTAG | GCTGGCATTGGGCTGTAGA |
| IL-7 | TTGGACTTCCTCCCCTGATC | TCGATGCTGACCATTAGAACA |
| IL-24 | TTGCCTGGGTTTTACCCTGC | AAGGCTTCCCACAGTTTCTGG |
| SOX6 | GGATGCAATGACCCAGGATTT | TGAATGGTACTGACAAGTGTTGG |
| SOD2 | GCTCCGGTTTTGGGGTATCTG | GCGTTGATGTGAGGTTCCAG |
| TP53 | AACTGCGGGACGAGACAGA | AGCTTCAAGAGCGACAAGTTTT |
| LIF | CCAACGTGACGGACTTCCC | TACACGACTATGCGGTACAGC |
| ABCG1 | ATTCAGGGACCTTTCCTATTCGG | CTCACCACTATTGAACTTCCCG |
| GAPDH | GGAGCGAGATCCCTCCAAAAT | GGCTGTTGTCATACTTCTCATGG |

Supplementary Table 1: The primers for qRT-PCR.
